# Parietal Lobe Epilepsy Associated With 2q13 Duplication: Expanding the Neurogenetic Spectrum

**DOI:** 10.7759/cureus.90456

**Published:** 2025-08-19

**Authors:** Christian Messina, Mariateresa Zuccarello

**Affiliations:** 1 Neurology, Azienda Sanitaria Provinciale Catania, Catania, ITA; 2 Neurology, University of Catania, Catania, ITA

**Keywords:** 2q13 duplication, duplication syndrome, eeg, electroencephalography, focal epilepsy, parietal lobe

## Abstract

Copy number variations (CNVs) involving the 2q13 region have been associated with a wide range of phenotypes, including developmental delay, dysmorphic features, hypotonia, and congenital heart defects. However, parietal lobe epilepsy has not yet been reported in association with 2q13 duplication. We describe the case of a 20-year-old woman with a duplication of the 2q13 region identified during childhood, who later presented with recurrent, brief episodes of right upper limb paresthesia spreading to other limbs, followed by transient pain. Her past medical history included dyspraxia, polycystic ovary syndrome, atrial fibrillation, and intellectual disability. Electroencephalography (EEG) revealed interictal epileptiform discharges, consisting of paroxysmal sharp theta waves in the left parietal and temporal regions, spreading to adjacent and contralateral areas, particularly during hyperventilation. Based on clinical and EEG features, a diagnosis of parietal lobe epilepsy was established. Treatment with levetiracetam resulted in significant clinical improvement, characterized by complete resolution of the previously described episodes of recurrent, brief right upper limb paresthesia spreading to other limbs, followed by transient pain, as well as a reduction of epileptiform abnormalities on EEG. The duplicated region includes several genes involved in neuronal development, synaptic regulation, and myelination, such as *MERTK*, *TMEM87B*, and *FBLN7*. Their altered expression may contribute to cortical excitability and epileptogenesis. This case adds to the phenotypic variability reported in individuals with 2q13 duplication and underscores the importance of further studies to explore possible gene-specific contributions to neurological findings. This is the first report linking 2q13 duplication with parietal epilepsy, underlining the importance of considering CNVs in unexplained focal epilepsy presentations.

## Introduction

Copy number variation (CNV) refers to a genomic phenomenon in which segments of DNA are repeated, with the number of copies varying among individuals of the same species [1]. CNVs can include duplications or deletions of genomic regions and may involve one or several genes [1]. While many CNVs are benign and present in the general population without clinical consequence, others have been implicated in a range of neurodevelopmental, neuropsychiatric, and neurological disorders [1]. Interpreting the clinical significance of novel or rare CNVs remains challenging, particularly when they involve genes with unclear dosage sensitivity or occur in genomic regions not yet well characterized [1]. A large number of novel CNVs have been identified in individuals with various clinical phenotypes, many of which are associated with specific conditions [1]. CNVs that are not reported in control population databases, do not overlap with regions known to be linked to diseases, and do not involve genes with established dosage sensitivity, are temporarily classified as variants of uncertain significance (VUS) [2]. Establishing a causal relationship between a novel CNV and a clinical phenotype remains both crucial and challenging [1,2]. Among these CNVs, duplications involving the long arm of chromosome 2, specifically the 2q13 region, have increasingly drawn attention due to their association with a wide and heterogeneous spectrum of clinical features [3]. This region contains multiple genes involved in key neurodevelopmental pathways, and duplications at this locus have been linked to a variety of phenotypes [3]. Reported features include dysmorphic traits, developmental delay, slender body habitus, fine motor planning deficits, abnormal head size, hypospadias, and plagiocephaly [3]. Although most of these manifestations are neurodevelopmental or structural in nature, seizures have also been described, albeit mainly in association with deletions of the 2q13 region [4,5]. To date, parietal lobe seizures have not been reported in individuals with 2q13 duplication. In this report, we aim to broaden the phenotypic spectrum of 2q13 duplications by describing a case of a woman with parietal epilepsy, thereby contributing to the growing body of evidence linking CNVs at this locus to variable neurological presentations.

## Case presentation

A 20-year-old woman was admitted to our clinic due to recurrent, transient episodes of paresthesia lasting approximately 30 seconds, initially affecting the right upper limb, then spreading to the ipsilateral lower limb and subsequently to the contralateral limbs. These episodes had started approximately two weeks before admission and had increased in frequency from about three to five times per day. Each episode was followed by localized pain in the affected regions, lasting several minutes. Her medical history was notable for childhood dyspraxia with impaired hand-eye coordination, mild intellectual disability, and a diagnosis of polycystic ovary syndrome in 2018. Birth history was unremarkable, with no perinatal asphyxia or complications. In 2022, she was diagnosed with atrial fibrillation and is currently under treatment with bisoprolol and flecainide. A genetic analysis performed in 2013 revealed a duplication involving the 2q13 chromosomal region. Family history included ischemic stroke in both the paternal and maternal grandfathers, and Parkinson’s disease in the paternal grandfather. Neurological examination revealed a prominent nose, dental crowding, brachydactyly, bilateral Hoffmann and Trömner signs, along with lateralized nystagmus to both the right and left. Blood tests, cerebrospinal fluid (CSF) analysis, carotid Doppler ultrasound, somatosensory evoked potentials (SEPs), visual evoked potentials (VEPs), and brainstem auditory evoked potentials (BAEPs) were unremarkable. Brain and spinal cord magnetic resonance imaging (MRI) were entirely normal, showing no relevant abnormalities and effectively ruling out any underlying structural pathology. Blood tests and CSF analysis are reported in Tables 1-2. Given the patient’s age and clinical presentation, testing for herpes simplex virus (HSV) and a comprehensive viral panel were also conducted to rule out viral infections that could mimic or precipitate demyelinating diseases such as multiple sclerosis, and to guide subsequent decisions regarding disease-modifying therapies. Anti-ganglioside antibody testing was included in the standard diagnostic work-up for patients presenting with unexplained sensory symptoms to investigate potential immune-mediated etiologies.

**Table 1:** Blood tests with their results during hospitalization Hb: hemoglobin; MCV: mean corpuscular volume; WBC: white blood count; CRP: C-reactive protein; LDH: lactate dehydrogenase; AST: aspartate aminotransferase; ALT: alanine aminotransferase; GGT: gamma-glutamyl transferase; CPK: creatine phosphokinase; TSH: thyroid-stimulating hormone; ANA: antinuclear antibodies; ENA: extractable nuclear antigens; IgM: immunoglobulin M; IgG: immunoglobulin G; HSV1: herpes simplex virus 1; HSV2: herpes simplex virus 2; VZV: varicella-zoster virus; CMV: cytomegalovirus; EBV: Epstein-Barr virus; VCA: antiviral capsid antigen; VDRL: venereal disease research laboratory; FTA-ABS: fluorescent treponemal antibody absorption; IGRA: interferon-gamma release assay

Lab tests	Results	Normal values
Hb	11.7 g/dL	10.5-13.5 g/dL
MCV	92 fL	80-100 fL
WBC	6.8 × 10^9^/L	4.0-11.0 × 10^9^/L
Neutrophils	3.7 × 10^9^/L	1.8-7.5 × 10^9^/L
Lymphocytes	2.1 × 10^9^/L	1.0-4.0 × 10^9^/L
CRP	0.9 mg/L	<5 mg/L
LDH	136 U/L	135-225 U/L
Sodium	140 mmol/L	135-145 mmol/L
Potassium	3.8 mmol/L	3.5-5.3 mmol/L
Glucose	75 mg/dL	69-105 mg/dL
Urea	3.2 mmol/L	2.5-7.8 mmol/L
Creatinine	0.72 mg/dL	<1.10 mg/dL
AST	23 U/L	8-33 U/L
AST	27 U/L	15-40 U/L
GGT	123 U/L	8-61 U/L
Lipase	35 U/L	14-72 U/L
Total amylase	68 U/L	30-118 U/L
CPK	56 U/L	29-168 U/L
TSH	3.7 μU/mL	2-10 μU/mL
ANA	1:20	<1:80
ENA screening	Negative	Negative
Anti-gangliosides IgM antibodies	Negative	Negative
Anti-gangliosides IgG antibodies	Negative	Negative
HSV1 IgM	2.9 U/mL	<20 U/mL
HSV1 IgG	28 U/mL	<20 U/mL
HSV2 IgM	0.5 U/mL	<20 U/mL
HSV2 IgG	9.5 U/mL	<20 U/mL
VZV IgM	9.2 U/mL	<25.5 U/mL
VZV IgG	26.2 U/mL	<25.5 U/mL
CMV IgM	0.5 U/mL	<18 U/mL
CMV IgG	2.3 U/mL	<18 U/mL
EBV VCA IgM	22 U/mL	<20 U/mL
EBV VCA IgG	12 U/mL	<35.9 U/mL
Toxoplasma IgM	0.2 IU/mL	<9 IU/mL
Toxoplasma IgG	0.1 IU/mL	<9 IU/mL
VDRL	Negative	<1:16
FTA-ABS	Negative	Negative
IGRA	Negative	Negative

**Table 2:** CSF analysis CSF: cerebrospinal fluid; IgG: immunoglobulin G; OCBs: oligoclonal bands

CSF analysis	Results	Normal values
Proteins	210 mg/L	135-500 mg/L
Glucose	59 mg/dL	<75 mg/dL
Cells	2/µL	<5/µL
IgG	15 mg/dL	-
IgG index	0.22	<0.7
OCBs	No OCBs	No OCBs
Anti-gangliosides IgM antibodies	Negative	Negative
Anti-gangliosides IgG antibodies	Negative	Negative

**Figure 1:**
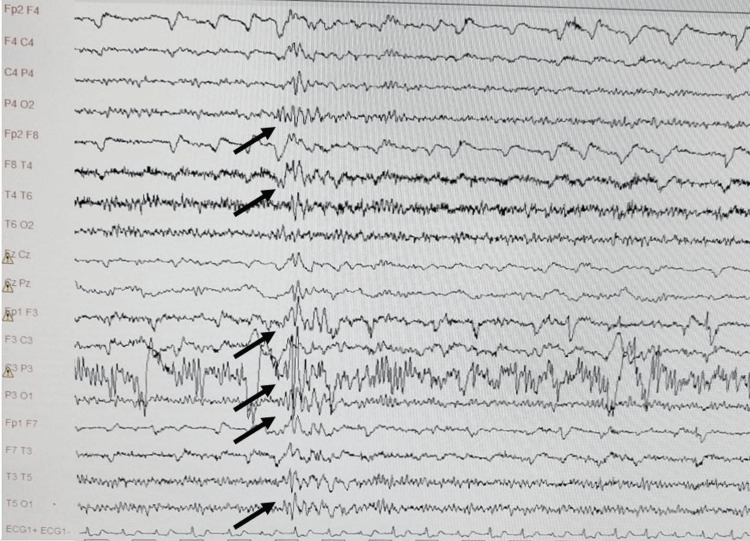
Electroencephalogram at hospital admission showing a discharge of sharp theta waves over the left parietal and temporal leads, with spread to adjacent and contralateral areas.

However, the electroencephalogram (EEG) revealed trains of sharp theta waves predominantly in the left parietal and temporal regions, with propagation to adjacent and contralateral areas (Figure 1). These abnormalities were accentuated during hyperventilation.

**Figure 2:**
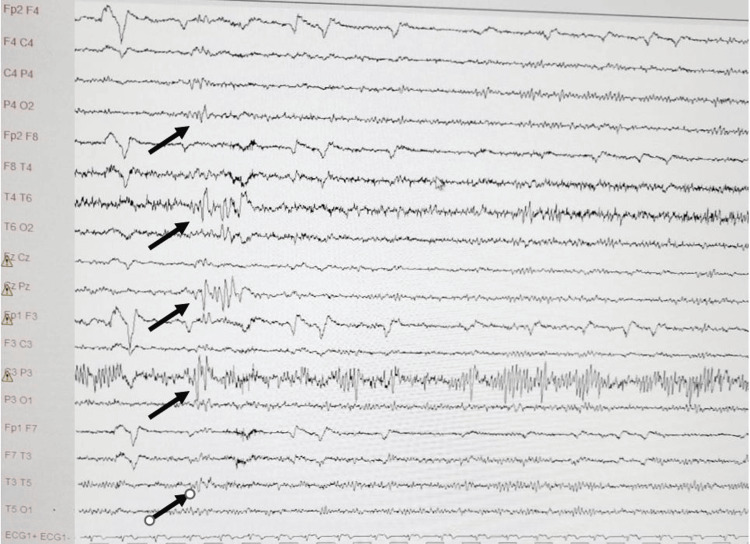
Electroencephalogram after ten days of treatment with Levetiracetam showing a reduction of sharp theta wave discharges in the left parietal and temporal regions, as well as in the adjacent and contralateral areas.

Based on these findings, a diagnosis of focal epilepsy originating from the left parietal lobe was established. The patient was started on levetiracetam 500 mg, one tablet twice daily. Ten days later, a follow-up EEG showed a reduction in epileptiform discharges in the left parietal and temporal regions, with decreased spread to adjacent and contralateral areas (Figure 2).

## Discussion

This is the first reported case of parietal lobe epilepsy associated with a 2q13 duplication. The EEG findings, showing interictal epileptiform discharges localized to the left parietal and temporal regions with propagation to adjacent and contralateral areas, provide a plausible electrophysiological explanation for the patient’s transient episodes of paresthesia initially affecting the right upper limb and subsequently spreading to other limbs. These clinical and neurophysiological correlations support the diagnosis of focal epilepsy originating from the parietal lobe. Previous studies have primarily focused on deletions and duplications within the 2q13 region as contributors to a broad phenotypic spectrum characterized by developmental delay, intellectual disability, and dysmorphic features [3]. Several authors have suggested that the 2q13 CNV may act as a susceptibility factor rather than a deterministic cause, influencing neurodevelopmental pathways in a variable and possibly dosage-dependent manner [3,4]. The exact mechanisms by which this CNV contributes to neurological phenotypes remain poorly understood, partly due to the heterogeneity of gene content within the duplicated region and the variable expressivity observed in affected individuals. Our report extends the clinical spectrum associated with 2q13 duplication to include parietal lobe epilepsy, highlighting the need for increased awareness of potential seizure disorders in patients harboring this genetic anomaly. It also underscores the importance of comprehensive neurophysiological assessment in individuals with CNVs presenting with transient neurological symptoms, to facilitate early diagnosis and appropriate management. The duplicated region can encompass up to seven genes (*BUB1*, *BCL2L11*, *MIR4435*-2HG, *ANAPC1*, *MERTK*, *TMEM87B*, and *FBLN7*), many of which are involved in cell cycle regulation and cellular homeostasis, potentially impairing neuronal function [4,5]. Recent knockdown studies in zebrafish have highlighted *FBLN7* and *TMEM87B* as candidate genes contributing to the 2q13 deletion phenotype [4,5]. Heterozygous loss of *FBLN7* has been associated with craniofacial abnormalities, as its depletion in zebrafish induces significant head and facial defects [4,5]. Moreover, individual or combined knockdown of *FBLN7* and *TMEM87B* has been shown to reproduce cardiac abnormalities, suggesting that loss of these genes may underlie the congenital heart defects and craniofacial dysmorphisms observed in 2q13 deletion syndrome [4,5]. Additionally, *FBLN7* and *TMEM87B*, alone or in combination with other genes in the region, may also be involved in neurological features such as developmental delay - phenotypes that are more difficult to assess in zebrafish models [4,5]. Moreover, the 2q13 duplication, involving the same aforementioned genes, has been reported in individuals with developmental delay and a range of phenotypic features, including dysmorphic traits (such as prominent nose, dental crowding, and hypertelorism), skeletal anomalies (including mild microcephaly, scoliosis, and clinobrachydactyly), schizophrenia, neurological impairments (notably hypotonia), and, less frequently, congenital cardiac defects [3]. These clinical features are in line with previously reported cases of 2q13 duplication, particularly with respect to brachydactyly, dental crowding, prominent nasal structure, early-onset cardiac disease (such as atrial fibrillation), intellectual disability, and dyspraxia. This overlap further supports the potential pathogenic relevance of the duplication in our patient’s phenotype. However, the woman described in this case report presents with focal epilepsy originating from the parietal lobe, a feature that has not previously been reported in association with 2q13 duplication. This novel presentation invites speculation about the underlying pathophysiology of epilepsy, which may be linked to the functional roles of the genes involved in this CNV. For example, enhancement of *MERTK* expression or function in the brain is likely to increase phagocytosis of its targets, including myelin, synapses, apoptotic cells, and even viable neurons, through a process known as phagoptosis [6]. This mechanism suggests that *MERTK* plays a key role in brain development, homeostasis, synaptic plasticity, and possibly in disease pathogenesis [6]. Therefore, gene dosage imbalance due to 2q13 duplication might lead to aberrant *MERTK* activity, contributing to altered neuronal function and potentially facilitating epileptogenesis through inappropriate synaptic pruning or neuronal loss. The *ANAPC1* gene, which encodes a core component of the anaphase-promoting complex/cyclosome (APC/C), is involved not only in cell cycle regulation but also in processes such as senescence, quiescence, DNA replication and repair, cellular differentiation, and neuronal function [7]. In the nervous system, APC/C plays a crucial role in maintaining cell cycle exit during neuronal differentiation [8]. Alterations in its activity - such as those potentially induced by gene dosage imbalance in 2q13 duplication - could result in neuron-specific cytokinesis defects. These defects may impair proper axonal development and function, suggesting a possible contribution to the neurological phenotype observed in our case, including parietal epilepsy and cognitive-motor symptoms. Similarly, the loss of *TMEM87B* expression has been shown to impair cellular viability by reducing cell proliferation and migration [9]. These functions are particularly relevant in neurodevelopment, where proper neuronal migration and proliferation are essential for cortical organization and connectivity [9]. *FBLN7* belongs to the fibulin family, a group of extracellular matrix glycoproteins found in both plasma and tissue, and is specifically expressed in oligodendrocyte-lineage cells and neuronal axons [10]. It plays a key role in oligodendrocyte differentiation and axon myelination, processes that are critical for the proper development and function of the central nervous system [10]. Disruptions in *FBLN7* expression or dosage, as may occur with 2q13 duplication, could therefore contribute to altered myelination and neurodevelopmental abnormalities. Further studies are needed to investigate whether altered dosage of genes within the 2q13 region may contribute to epileptogenesis by disrupting normal neuronal development, synaptic function, or network excitability in specific brain areas, such as the parietal lobe. Understanding the molecular and cellular mechanisms through which these gene duplications influence neural circuitry could provide valuable insights into the pathophysiology of focal epilepsies associated with CNVs. Additionally, elucidating gene-specific effects may help identify potential biomarkers for risk stratification and guide targeted therapeutic interventions. Given the complexity and variability of CNV-related phenotypes, large-scale genetic and functional studies are essential to clarify genotype-phenotype correlations and improve clinical management of affected individuals.

## Conclusions

This case contributes to the expanding phenotypic spectrum associated with 2q13 duplication by reporting, for the first time, parietal lobe epilepsy in a carrier of this CNV. It emphasizes how genetic alterations such as rare CNVs can be linked to a broader range of clinical presentations than those traditionally recognized. This underlines the importance of including rare CNVs in the differential diagnosis of atypical neurological manifestations, especially in cases where common causes have been excluded, given their variable expressivity and potential to produce phenotypes beyond the classical descriptions found in the literature. The genes within the duplicated 2q13 region are known to be involved in critical neurodevelopmental and synaptic functions, suggesting a possible association with the observed epileptic phenotype. These genes may influence neuronal connectivity, synaptic plasticity, or network excitability, factors that are relevant to the pathophysiology of epilepsy. However, the exact role and contribution of each gene remain to be fully elucidated. Further research, including functional studies and larger patient cohorts, is necessary to investigate the specific contributions of these genes and to better understand the mechanisms by which such genomic alterations may be related to epileptogenesis and other neurological features. This case report thus highlights the value of comprehensive genetic analysis in patients with unusual neurological presentations and supports ongoing efforts to refine genotype-phenotype correlations in CNV-related disorders. Increased awareness and reporting of such cases will be essential to improve diagnostic accuracy and ultimately guide patient management and counseling.
